# Evaluation of Natural and Modified Castor Oil Incorporation on the Melt Processing and Physico-Chemical Properties of Polylactic Acid

**DOI:** 10.3390/polym14173608

**Published:** 2022-09-01

**Authors:** Raluca Nicoleta Darie-Niță, Anamaria Irimia, Vasile Cristian Grigoraș, Fănică Mustață, Niță Tudorachi, Maria Râpă, Joanna Ludwiczak, Andrzej Iwanczuk

**Affiliations:** 1 Physical Chemistry of Polymers Department, Petru Poni Institute of Macromolecular Chemistry, 41A Grigore Ghica Voda Alley, 700487 Iasi, Romania; 2 Natural Polymers, Bioactive and Biocompatible Materials Department, Petru Poni Institute of Macromolecular Chemistry, 41A Grigore Ghica Voda Alley, 700487 Iasi, Romania; 3 Faculty of Materials Science and Engineering, University Politehnica of Bucharest, 313 Splaiul Independentei, 060042 Bucharest, Romania; 4 Faculty of Environmental Engineering, University of Science and Technology, 50-013 Wrocław, Poland

**Keywords:** poly(lactic acid), castor oil, plasticizer, mechanical properties, rheology, thermal properties

## Abstract

Bio-based plasticizers derived from renewable resources represent a sustainable replacement for petrochemical-based plasticizers. Vegetable oils are widely available, non-toxic and biodegradable, resistant to evaporation, mostly colorless and stable to light and heat, and are a suitable alternative for phthalate plasticizers. Plasticized poly(lactic acid) (PLA) materials containing 5 wt%, 10 wt%, 15 wt% and 20 wt% natural castor oil (R) were prepared by melt blending to improve the ductility of PLA. Three castor oil adducts with maleic anhydride (MA), methyl nadic anhydride (methyl-5-norbornene-2,3-dicarboxylic anhydride) (NA) and hexahydro-4-methylphthalic anhydride (HA), previously synthesized, were incorporated in a concentration of 15 wt% each in PLA and compared with PLA plasticized with natural R. The physico-chemical properties of PLA/R blends were investigated by means of processability, chemical structure, surface wettability, mechanical, rheological and thermal characteristics. The addition of natural and modified R significantly improved the melt processing by decreasing the melt viscosity by ~95%, increased the surface hydrophobicity, enhanced the flexibility by ~14 times in the case of PLA/20R blend and ~11 times in the case of PLA/15R-MA blend as compared with neat PLA. The TG/DTG results showed that the natural R used up to 20 wt% could significantly improve the thermal stability of PLA, similar to the maleic anhydride-modified R. Based on the obtained results, up to 20 wt% natural R and 15 wt% MA-, HA- or NA-modified R might be used as environmentally friendly plasticizers that can improve the overall properties of PLA, depending on the intended food packaging applications.

## 1. Introduction

Environmentally friendly plasticizers derived from renewable resources are considered sustainable substitutes for petrochemical-based plasticizers. The use of vegetable oils as an alternative to fossil raw materials for preparing chemical products is regarded as a modern challenge from both an environmental and economic point of view. Due to their wide availability, non-toxicity and biodegradability, resistance to evaporation, and being mostly colorless and stable to light and heat, vegetable oils are a suitable alternative for phthalate plasticizers [1,2].

Castor oil, also known as ricinum oil (R), produced by pressing from castor (*Ricinus communis*) beans, is a multipurpose vegetable oil used since ancient times, considered a vegetable oil due to the high amount of monounsaturated fatty acid and bioactive compounds. The fatty acid profile comprises mainly ricinoleic acid and other minor acids such as stearic, oleic, and palmitic acid. Triricinolein constitutes the predominant triglyceride component in the oil. Minor biological compounds that confer oxidation stability, anti-inflammatory, and antioxidant properties to R include carotenoid, tocopherol, tocotrienol, phospholipid, phytosterol, phytochemical, and phenolic compounds [3].

**Scheme 1:**
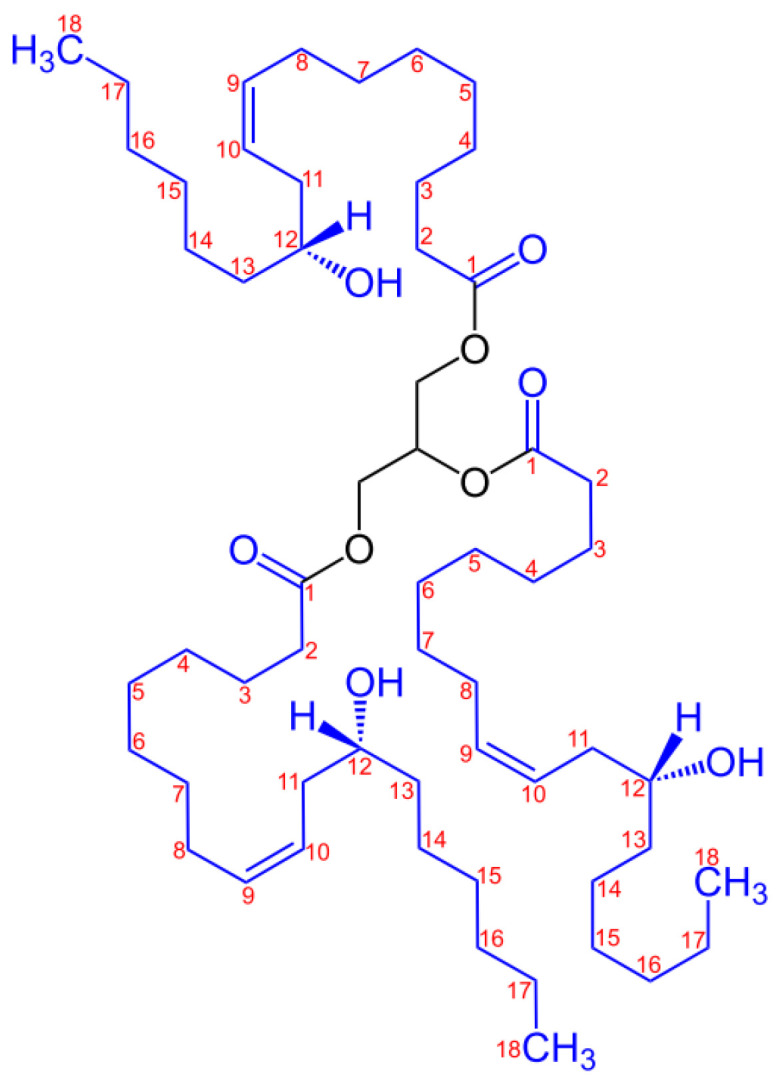
Structure of the major component of castor oil: triester of glycerol and ricinoleic acid [14].

Castor oil has long been considered medicinal oil, especially for its purgative or laxative properties as a remedy for constipation [4]. Castor oil is classified as non-edible oil due to its nauseant properties [5]. Therefore, food-grade castor oil is only found in applications in the food industry in food additives, flavorings, and candy. The high value of castor oil is given by the high content of ricinoleic acid, thus finding multiple versatile application possibilities in the chemical industry. Hence, castor oil cannot compete with food sources due to its utilization as a plasticizer. Castor oil is compatible with many natural and synthetic resins, polymers, and waxes [6]. The European Union approves it for food contact use without any restrictions. R has found applications as a raw material for a variety of polymers such as polyamides (i.e., Nylon-11), [7] polyurethanes [8,9] or interpenetrating networks [10,11]. The hydrogenated R derived from 12-hydroxystearic castor oil, glycerin and acetic acid was used as plasticizer for poly(vinyl chloride) processing [12,13]. R is unique because it carries a special ricinoleic acid, which has an 18-carbon backbone with a hydroxy group on the 12-carbon atom and a cis double bond between carbons 9 and 10 (Scheme 1). 

The hydroxyl in the R molecule can undergo esterification to obtain more polar groups to improve its compatibility with polar materials such as PVC and nitrile rubber (NBR) [15]. In another study, unmodified and modified R were employed to increase the reactivity towards diphenylmethane diisocyanate (MDI), a component used to process bio-based polyurethanes [16]. The pyrolysis process of R led to the valuable synthesized carbon nanorods (CNR), which has application for detecting volatile biomarkers [17].

Poly(lactic acid) (PLA) is a thermoplastic polymer obtained from renewable resources showing several benefits such as biodegradability, biocompatibility and compostability. The Food and Drug Administration has authorized this environmentally friendly polymer for human use in biomedical and food-contact applications. PLA and its blends and composites have been extensively used in food packaging [1,18,19], medical applications [20,21,22], and environmental protection [23,24]. Although PLA shows good processability, high tensile strength and stiffness, it has poor thermal, flexibility, impact strength and barrier properties that limit PLA end-use application [25]. In order to overcome the limitations associated with PLA, strategies like blending of PLA with different plasticizers such as acetyl tri-n-butyl citrate (ATBC), poly(ethylene glycol) (PEG) [26,27], acetyl tris(2-ethylhexyl) citrate, tris(2-ethylhexyl) citrate, and poly(ethylene glycol)bis(2-ethylhexanoate) [28] or other polymers [29,30,31,32] or incorporation of functional filler agents [25,33] in the PLA matrix are proposed. 

More attention is being paid to environmental, biodegradable and low-toxic vegetable oil-based plasticizers used in polyesters. Literature data revealed few studies relating to modifying PLA properties with R. For example, PLA, talc and 1% R were used to prepare dental mouth mirror products by injection molding technology [34]. It was found that the R helps to increase the impact strength up to 56% due to the improved adhesion between the PLA and talc. In another paper, unmodified R was used for covering PLA pellets, which were further combined with lignin with an application for the healthcare field by 3D printing [35]. The results showed that the R did not influence the contact angle values of the materials. Robertson et al. [36] mentioned that the addition of 5 wt% R to poly(L-lactide) (PLLA) showed no plasticization of the PLLA, although it significantly enhances the overall tensile toughness. The authors synthesized poly(ricinoleic acid)-PLLA diblock copolymers and used them as compatibilizers for the PLLA/R blends [36]. 

The novelty of the current work lies in investigating the melt processing, structural modifications, surface wettability, dynamic melt rheology, and mechanical and thermal stability of PLA blended with 5, 10, 15 and 20 wt% natural R. We compared it with PLA containing 15 wt% R adducts with maleic anhydride (MA), methyl nadic anhydride (methyl-5-norbornene-2,3-dicarboxylic anhydride) (NA) and hexahydro-4-methylphthalic anhydride (HA), respectively, previously synthesized and characterized by two of the co-authors. This approach has not been documented earlier to the best of our knowledge. The modified castor oils were used as a strategy to enhance the hydrophobic character and overall mechanical properties related to the same amount of natural castor oil and to evaluate the differences between the types of cyclic anhydrides on the rheological, mechanical and thermal performance of PLA/modified castor oil materials.

## 2. Materials and Methods

### 2.1. Materials

PLA Ingeo^TM^ Biopolymer 2003D supplied by NatureWorks LLC (Minnetonka, MN, USA), having a specific gravity of 1.25 g·cm^−3^, Mw of 1.43 × 10^5^ g·mol^−1^, Mn of 7.54 × 10^4^ g·mol^−1^ and dispersity index (*Đ*_M_) of 1.88 was used in this study.

Castor (*Ricinus communis*) oil (R) is a commercial product containing 90% ricinoleic acid, with a density of 950 kg m^−3^, iodine value of 83 g I_2_ 100 g^−1^, a hydroxyl value of 161 mg g^−1^, purchased from the local market (Herbavit, Romania). 

**Scheme 2:**
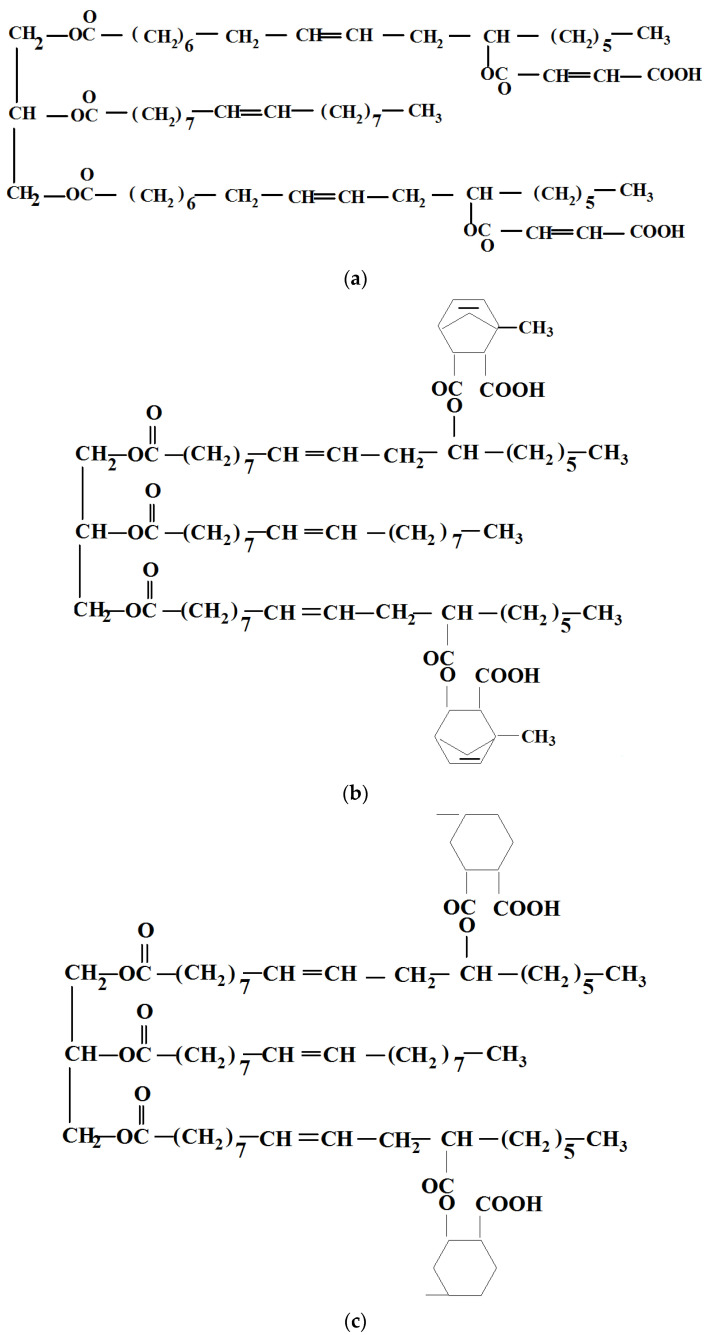
The chemical structures of (**a**) R-MA, (**b**) R-NA, and (**c**) R-HA.

Castor oil, a triglyceride ester of ricinoleic acid containing up to 2.7 hydroxyl groups per molecule, was converted with three cyclic anhydrides into esters with carboxyl in their structures by Mustata and Tudorachi [37]. Three castor oil adducts with maleic anhydride (MA) (R-MA), methyl nadic anhydride (methyl-5-norbornene-2,3-dicarboxylic anhydride) (NA) (R-NA) and hexahydro-4-methylphthalic anhydride (HA) (R-HA) (Scheme 2) were synthesized in the presence of stannous octoate as a catalyst, at a molar ratio 1/2.7 (oil/anhydride), reaching conversions greater than 99 wt% in medium temperature conditions. The modified vegetable oils were further characterized, the acid values being 130 mg g^− 1^ for R-MA, 132 mg g^− 1^ for R-NA and 134 mg g^− 1^ for R-HA.

### 2.2. Sample Preparation

Before processing, PLA pellets were dried at 60 °C for 24 h in a vacuum oven to remove moisture that might lead to degradation of macromolecular chains (moisture content < 200 ppm). The melt processing of the PLA with natural R and modified R (R-MA, R-NA and R-HA) was realized during melt mixing for 8 min at a temperature of 175 ± 5 °C using a Thermo Scientific PolyLab QC mixer provided with a mixing chamber of 50 cm^3^, the screws speed of counter-rotating rotors being of 60 rotations/minute. The homogenized melted mixtures were hot-pressed at 175 °C on a laboratory LabTech LP-20B hydraulic press (LabTech, Samut Prakan, Thailand) using a preheating for 5 min at 100 bar and a pressing step for 3 min, at a pressure of 147 bar, followed by cooling in order to obtain thin homogeneous plates with dimensions of 150 × 150 × 1 mm. Specimens were prepared from these plates for testing of tensile (‘dog bone’ shape), Fourier transform-infrared spectroscopy (FT-IR), contact angle, differential scanning calorimetry (DSC), thermogravimetry (TG), and dynamic rheology. The compositions, visual aspects of 1 mm thickness samples and labeling of the resulting materials based on PLA and plasticizers are presented in Table 1. All obtained materials were homogeneous and transparent, with a very low tendency of opacity for PLA-based materials containing modified R but no yellowness due to the thermal degradation during processing and pressing steps. A white paper with a black line was used as the background to observe the transparency better.

### 2.3. Investigation Methods

#### 2.3.1. Melt Processability

The processing behavior of neat PLA and PLA samples containing modified and unmodified R was evaluated by monitoring the processing characteristics from the torque-time curves recorded during blending in the Thermo Scientific PolyLab QC mixer for 8 min. Since the torque is proportional to the shear stress (SI unit: Pa or N m^−2^), and the rotor speed is proportional to the shear rate (SI unit: s^−1^), the ratio between torque to the rotor speed was the approximate melt viscosity (SI unit: N·s m^−2^ or Pa·s or kg·m^−1^·s^−1^), which was calculated from the ratio of torque recorded at 8 min, expressed in Nm to the fixed rotor speed of 60 rpm, according to Equation (1) [38]. The power consumption (P) is calculated by the multiplication of torque at the end of mixing (TQ_fin_) with rotor speed—Equation (2) [38].

η = K(TQ_fin_/S) (Pa·s) (1)

P = TQ_fin_ × 2πS/60 (W) (2)

where: K is a constant dependent on temperature, TQ_fin_ is the torque at the end of processing time (N·m), and S refers to the rotor speed (rotations/minute, rpm). 

#### 2.3.2. Attenuated Total Reflection-Fourier Transform Infrared Spectroscopy (ATR-FTIR)

The infrared spectra were recorded in ATR mode using a Brucker ALPHA (Platinum ATR) FTIR spectrometer (Bruker Optics, Ettlingen, Germany) equipped with a diamond crystal in the 3050–3600 cm^−1^ region, with a resolution of 4 cm^−1^ using air as background, all spectra representing the average of 20 scans. Three recordings were performed for each sample, and the evaluation was made on the average spectrum obtained from these recordings. The processing of the spectra has been done with OPUS 7.5 program. Prior to each test, a background spectrum was obtained to compensate for the humidity effect and the presence of carbon dioxide by spectra subtraction. 

#### 2.3.3. Water Contact Angle Measurements

Water contact angle (WCA) measurements were used to determine the influence of R incorporation over the hydrophobicity of the PLA surface. The wettability of surfaces was determined by static contact angle measurements performed on a CAM-200 goniometer (KSV Instruments Ltd., Helsinki, Finland). The water contact angle was determined by the sessile drop method, at room temperature and controlled humidity, within 5 s after placing 1 µL drops of liquid on the sample’s surface. A video camera recorded the evolution of the droplet shape, and image analysis software was used to determine the contact angle values. At least 10 measurements were performed on a sample, and results from three different samples were considered for statistical determination of the final average value of a material.

#### 2.3.4. Stress-Strain Measurements

Tensile properties such as tensile strength, elongation at break and Young’s modulus were determined according to EN ISO 527-2:2011 using the Lloyd LR10K machine (Lloyd. Instruments Ltd., Bognor Regis, UK) on “dog bone” specimens of 1 mm thickness and 40 mm length taken from plates obtained by hot-pressing. The stretching of the samples took place using a load cell of a maximum of 500 N at a crosshead speed of 10 mm min^−1^. At least seven specimens were measured for each composition, and the average value was reported. The specimens were conditioned under the same conditions for 24 h before testing. All mechanical tests took place at 50% relative humidity and 23 °C.

#### 2.3.5. Dynamic Rheology

Oscillatory frequency tests were performed in melt state at 175 °C using an Anton Paar rheometer (MCR301, Graz, Austria) equipped with CTD450 in parallel-plate geometry (diameter of 25 mm). The oscillatory frequency sweeps ranged from 0.05 to 500 rad/s, with a constant strain of 10% (falling in the linear domain of viscoelasticity). The gap between the parallel plates used during testing has been set to 1 mm.

#### 2.3.6. Differential Scanning Calorimetry (DSC)

The thermal properties of the neat and plasticized PLA samples were determined using a TA Instruments Q20 differential scanning calorimeter (New Castle, DE, USA) under a nitrogen atmosphere. The samples were firstly heated at a rate of 10 °C min^−1^ from 25 °C to 200 °C, held for 2 min, and then cooled down to 25 °C with a cooling rate of 5 °C min^−1^. This was followed by the second heating run from 25 °C to 200 °C at a heating rate of 10 °C min^−1^. Nitrogen was used as a furnace purge gas. A sample mass of ~7 mg of each material was tested; triplicate samples were analyzed by DSC. Thermal parameters such as the glass transition temperature (Tg), melt temperature (Tm), cold crystallization temperature (Tcc), and enthalpy of melting (Δ*H*_m_) were determined from DSC spectra.

The crystallinity of the studied materials was calculated by applying Equation (3) [39], where Δ*H*_m_ refers to the enthalpy of melting, Δ*H*cc is the enthalpy of cold crystallization, *w*_PLA_ is the weight percentage of PLA in the samples and $\Delta H_{m}^{0}$ is the enthalpy of melting for 100% crystalline PLA, with a value of 93.7 J/g [40].

(3) $$\chi_{c}=\frac{\Delta H_{m}-\Delta H_{\mathrm{cc}}}{{\Delta H}_{m}^{0}*w_{\mathrm{PLA}}}*100\%$$

#### 2.3.7. Thermogravimetric Analysis (TG/DTG)

The thermal degradation was evaluated using a thermogravimetric balance model STA 449 F1 Jupiter (Netzsch, Selb, Germany). Temperature and sensitivity calibration was performed with standard metals (In, Sn, Bi, Zn, Al). Sample weights in the 7 to 10 mg range were placed in Al_2_O_3_ crucibles and heated from 25 °C to 700 °C with a heating rate of 10 °C min^−1^, Al_2_O_3_ being considered reference material. The nitrogen (99.999% purity) was used as a purge and protective gas with a flow rate of 40 mL min^−1^. Data collection was carried out with Proteus^®^ software.

## 3. Results and Discussions

The incorporation of 5 to 20 wt% of natural castor oil in PLA aims to improve the melt flow of the semicrystalline matrix, with a benefit on the melt processing of the resulting materials, together with the enhancing effect on the flexibility and thermal stability of brittle PLA, thus expanding its use in specific food packaging applications. Due to possible physical interaction between the PLA matrix and anhydride-containing plasticizers, we expect the modified castor oils to improve the hydrophobicity and overall mechanical properties, both regarding elongation but also inducing a reinforcement effect compared with PLA/natural R, with a different result over the thermal stability and decomposition of blended materials function of the used anhydride.

### 3.1. Evaluation of the Melt Processing 

The influence of R amounts incorporated into PLA over the melt processability has been evaluated by following the maxim torque (TQ_max_), torque after one minute (TQ_1min_) and five minutes of mixing (TQ_5min_), the final torque at the end of mixing (TQ_fin_), melt viscosity (η) and power consumption (P) (Table 2). The torque versus processing time curves were inserted in the Supplementary Materials (Figure S1). As already known, the neat PLA has a semicrystalline structure that represses the polymer chain motion. Therefore, high torques, melt viscosity and power values are recorded for the pristine PLA [18].

A constant value of torque was reached between 4 and 5 min of mixing for most of the samples, being related to the complete melting of the PLA matrix and the homogeneous mixing with the vegetal oil. The torques values of PLA/R samples obviously decreased compared with neat PLA due to the plasticizer action of incorporated R that enhances the chain mobility. By increasing the amount of R from 5 wt% to 20 wt%, an exponential decrease in processing characteristics was observed as the frictional resistance is reduced due to much easier deformation of the PLA chains, improving melt flow and, consequently, melt processability. As expected, PLA containing 20 wt% R presented a lower melt viscosity for PLA/unmodified R sample due to the low viscosity index of R [41]. A rise in the torques values after 5 min and at the end of mixing and respectively, melt viscosity and power consumed for processing has been observed for PLA/15R-MA and PLA/15R-HA when compared with PLA/15R possible due to the physical interaction between the PLA matrix and anhydride-containing plasticizers.

The melt processing behavior showed no occurrence of phase separation, indicating that unmodified and modified R are miscible with PLA, the melted material being easily removed from the rotors.

### 3.2. ATR-FTIR Results

The FTIR spectra were analyzed to describe the structural changes in the PLA materials before and after plasticization and determine the crystallinity indices.

Synthesis and characterization of R modified with three anhydrides used in this study were published in previous work [37]. 

**Figure 1:**
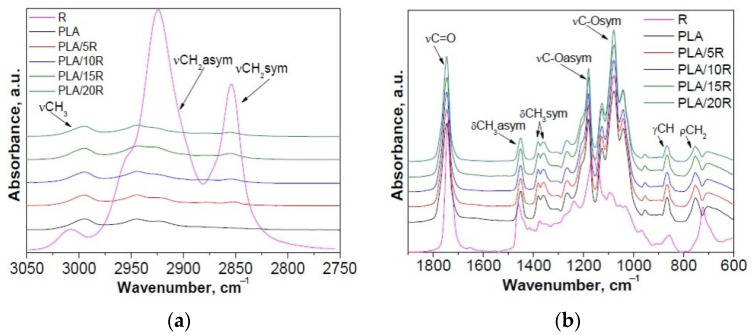
Normalized ATR-FTIR spectra of PLA/R samples compared with neat PLA and R: (**a**) 2750–3050 cm^−1^ and (**b**) 600–1900 cm^−1^ spectral range.

**Figure 2:**
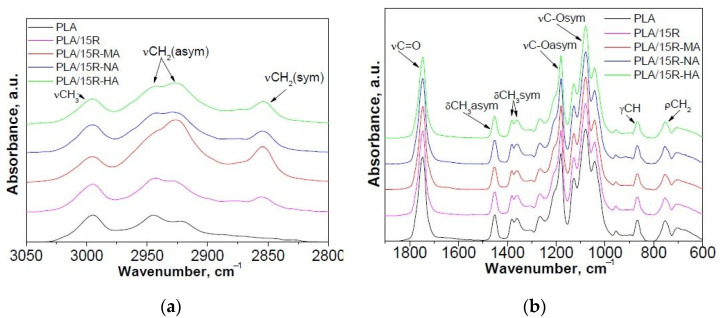
Normalized ATR-FTIR spectra of PLA/modified R samples compared with neat PLA and PLA/15R samples: (**a**) 2750–3050 cm^−1^ and (**b**) 600–1900 cm^−1^ spectral range.

Modification of PLA with R revealed changes in the 2700–3050 cm^−1^ region, assigned to the CH stretching vibrations, and in the “fingerprint” 600–1900 cm^−1^ region of the FTIR spectra, which corresponds to stretching or deformation vibrations of different groups (Figure 1 and Figure 2).

FTIR spectra of neat PLA displayed two bands at 2995 and 2943 cm^−1^ (corresponding to the asymmetric stretching vibration of C–H from CH_3_ and CH_2,_ respectively); a strong absorbance band at 1746 cm^−1^ (attributed to the stretching vibrations of amorphous C=O groups); bands at 1452 cm^−1^ and 1382 cm^−1^, 1359 cm^−1^ characteristic for asymmetric and symmetric bending vibration of C–H from CH_3_); strong absorbance bands at 1180 cm^−1^ (asymmetrical valence vibrations of C–O–C of the aliphatic chain) and 1080 cm^−1^ (symmetrical valence vibrations of C–O–C of the aliphatic chain). The band at 868 cm^−1^ can be assigned to the amorphous phase, while the band at 755 cm^−1^ to the crystalline phase [42,43].

In the case of PLA/R blends with different concentrations of R, a slight increase of the signal from 2943 cm^−1^, assigned to the CH_2_ group, asymmetric stretching vibration can be observed, while a new signal was detected at about 2855 cm^−1^ corresponding to the CH_2_ group, symmetric stretching vibration, from the main chain of ricinoleic acid (~90%), the main component from R (Figure 1a). Moreover, an increase in band intensity due to the overlapped vibration bands of PLA and R was recorded at 1746 cm^−1^ (C=O group, stretching vibrations). A decrease in intensity for bands from 1452 cm^−1^ and 1382 cm^−1^, 1358 cm^−1^ that corresponds to asymmetric and symmetric bending vibration of C–H from CH_3_ due to the decrease of CH_3_ moieties in PLA/R blends compared to neat PLA was observed (Figure 1b). Based on these results, the greatest changes were observed for PLA/15R, the blending efficiency being higher for PLA with 15 wt% R. In this sense, 15 wt% loading of R modified with different anhydrides was used for incorporation into the PLA matrix. 

The effect of incorporation of R modified with anhydrides on FTIR spectra of PLA is noticed in Figure 2. An increase of the signal from 2926 cm^−1^, assigned to the CH_2_ group, asymmetric stretching vibration, and a new signal at about 2855 cm^−1^ corresponding to the CH_2_ group, symmetric stretching vibration, was observed (Figure 2a). Moreover, a decrease in band intensity and a small shift from 954 cm^−1^ for PLA to 957 cm^−1^ PLA/15R-anhydrides (corresponding to the O-H vibration of carboxylic acid) is notable. This can be due to the Van der Waals interactions between PLA’s and anhydrides modified castor oil’s functional groups, which result in a hydrogen bridge connecting hydrogen from the hydroxyl group in PLA and oxygen from the carboxyl group of anhydrides modified R [44].

**Figure 3:**
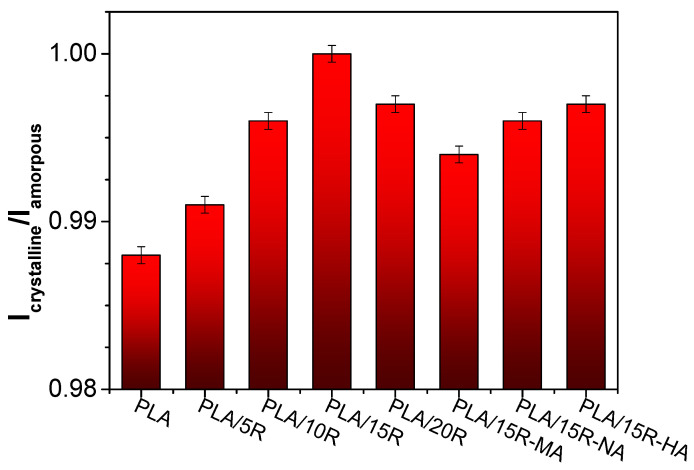
I_crystalline_/I_amorpous_ ratio derived from FTIR data.

The ratio between the normalized intensities of bands at 755 cm^−1^ and 868 cm^−1^ is proportional to the crystallinity index, and the corresponding values for studied samples are given in Figure 3. 

Although the variations in the I_crystalline_/I_amorphous_ ratio calculated from the FTIR spectra are small, they provide insight into the crystallinity indices calculated more precisely from DSC thermograms. A slight increase of the I_crystalline_/I_amorphous_ ratio from 0.988 in PLA up to 1.00 in PLA/15R is evident in the case of PLA/unmodified R blends. This indicates a modification in the structure of the systems containing plasticizers due to the increase in chain mobility. Similar findings have been reported in the literature [45,46,47].

The crystallinity indices of PLA/anhydrides modified R slightly decreased compared to PLA/R, from 1.00 in PLA/15R to 0.994 in PLA/15R-MA (Figure 3). The variation in the modification corresponds with the composition of modified R. It appears that the plasticization efficiency was higher for the sample containing HA- and NA- modified R, which recorded the highest crystallinity index values among the modified R. 

### 3.3. Water Contact Angle (WCA)

Water contact angle measurements evaluated the wettability of the developed materials on the surfaces of 1 mm thickness plates. If the contact angle of water is larger than 90°, the surface is considered hydrophobic, while for contact angles smaller than 90°, the surfaces are categorized as hydrophilic. The hydrophilic/hydrophobic balance influences the applications of PLA blends, such as films for food packaging [31] or medical fields involving drug delivery and tissue engineering scaffolds, where increased hydrophilicity is needed [48].

**Figure 4:**
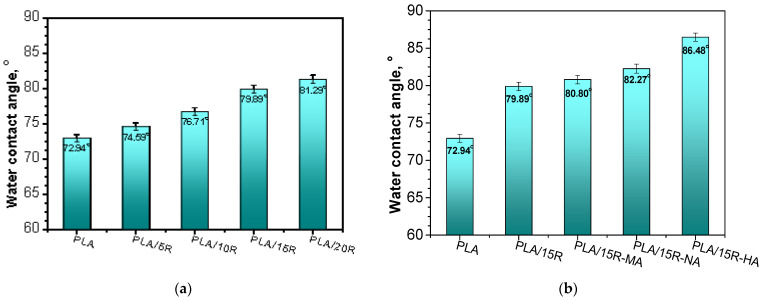
Water contact angle of (**a**) neat PLA and PLA-based materials containing pure castor oil; (**b**) PLA-based materials containing anhydrides modified castor oil.

Figure 4a,b presents the WCA of PLA mixtures with natural and modified R. Pristine PLA sample presented a WCA of 72.94°.

By blending PLA with natural R, an increase in WCA up to 81.29° was observed for the highest amount of incorporated R. Thus, by adding R as plasticizer up to 20 wt%, the hydrophobic character of the PLA was significantly enhanced. The hydrophobicity is attributed to the presence of ricinoleic acid, the major component of R (~90%) [49]. 

Furthermore, the WCA for PLA/anhydrides modified R samples was slightly higher compared with PLA/R samples (Figure 4). Using the modified R in PLA blends, the WCA increased from 79.89° in PLA/15R to 86.48° in PLA/15R-HA. This can be explained by the interactions that occur between the polar functional groups of the main component from R (e.g., hydroxyl) and different anhydrides [37], leading to fewer available groups at the top surface of PLA/modified R samples to interact with water, thus obtaining materials with lower surface wettability. The water contact angle values for samples containing R-HA and R-NA were slightly higher than those containing R-MA. This effect is likely due to the ability of longer side chains to create more intermolecular bonds, causing the decrease of the hydrophilic functional groups available on the surface of PLA blends [50]. A contrary effect of decreasing the hydrophobicity was reported in the case of the introduction of the poly(ethylene glycol) (PEG) plasticizer into PLA/PEG systems [27] due to its solubility in water. Another study [51] reported the increased hydrophobicity of food packaging materials by using modified cellulose. Hydrophobicity was known to ensure an improved water barrier, lower permeability to moisture, and mechanical durability of food packaging material [51]. 

### 3.4. Stress-Strain Results

**Figure 5:**
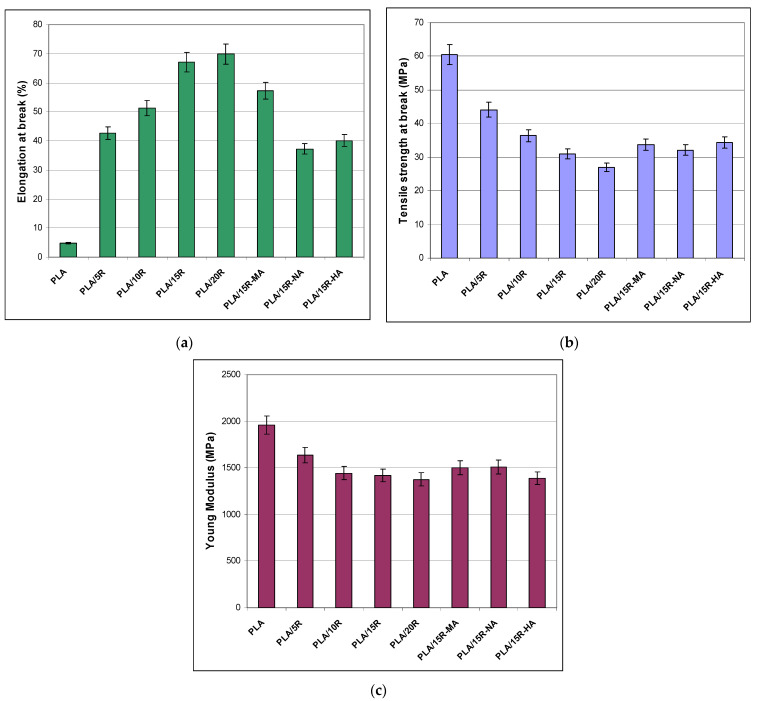
Mechanical properties for neat and plasticized PLA: (**a**) elongation at break, (**b**) tensile strength at break, (**c**) Young modulus.

The results of the tensile tests for the neat PLA and PLA plasticized with different contents of unmodified R and 15 wt% anhydride–modified R are summarized in Figure 5. PLA is characterized by a high Young modulus and brittleness, limiting its use in specific applications. The aim of incorporating plasticizers in the PLA matrix is to reduce brittleness and enhance flexibility. 

The PLA samples containing natural R display ~14 times increase of elongation at break, from a value of 4.7% for neat PLA up to 67% for PLA/15R and 70% for PLA/20R. A 10 times increase in elongation at break was reached even at 5 wt% natural R incorporated in the PLA matrix, while a relatively similar increment was observed for the highest amounts of unmodified R, 15 wt% and 20 wt%. This flexibility enhancement is associated with a gradual decrease of the tensile strength at break and Young modulus. The highest values of elongation at break recorded for PLA/15R and PLA/20R denoted good compatibility between PLA and R at these elevated loadings. The flexibility enhancement appeared because R increased the chain’s mobility by filling the space between the polymer chains, disrupting the intermolecular bonds between PLA chains (polymer-polymer interaction), followed by substitution with hydrogen bonds formed between plasticizer and polymer chains (plasticizer-polymer interaction). 

One can observe that incorporating R-MA in the PLA matrix led to the development of a more flexible material among the used cyclic anhydride—modified R, reporting a more than 11-fold increase of elongation at break related to neat PLA and almost double improvement when compared with R-NA. However, the mechanical results demonstrated a reinforcing effect of PLA blended with modified R in relation to PLA containing 15 wt% natural R, both strength at break and Young modulus values increasing. Overall, all obtained tensile properties recommend using PLA plasticized with unmodified R for flexible film packaging, while those containing modified R for semi-rigid food packaging. Lower values for elongation at break in the case of PLA/vegetal oils were reported in the literature. The incorporation of up to 20 wt% epoxidized sunflower oil (ESO) in PLA led to a slight increase of elongation to values of 9% for PLA/ESO 5.5%, to 16% for PLA/ESO 6.5%, and 34% for and PLA/commercial epoxidized soya bean oil [42]. Similar results to our findings for mechanical properties were published for other plasticized polyesters. Tensile tests of 3D printed samples (dogbones) measured for non-plasticized PHB-PLA blend (reference) and PLA/PHB blends plasticized with acetyl tris(2-ethylhexyl) citrate, tris(2-ethylhexyl) citrate, and poly(ethylene glycol)bis(2-ethylhexanoate) showed an increase of elongation at break from 10% (reference) to 32% for plasticized blends in the form of printed dogbones [28]. A significant increase in elongation at break was reported by Mencik et al. [30] in the case of plasticizing PHB/PLA with commercial esters of citric acid for Three-Dimensional (3D) print: from 5% in the case of reference to 187% for the printed dogbones of PHB/PLA/plasticizer blends. 

### 3.5. Dynamic Rheology

**Figure 6:**
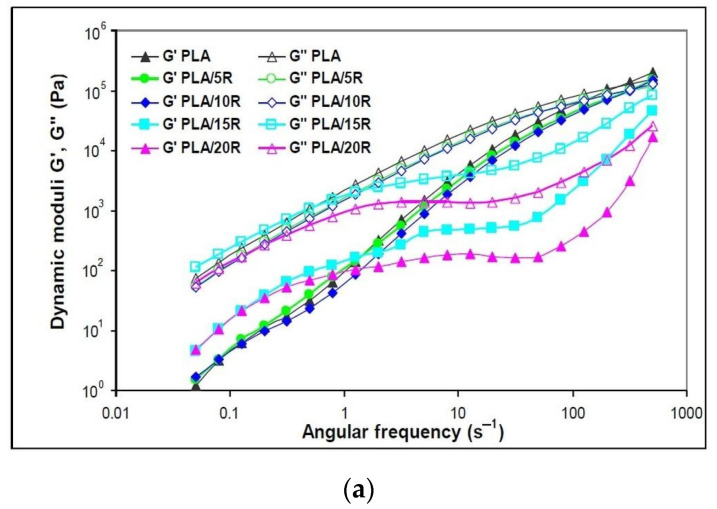

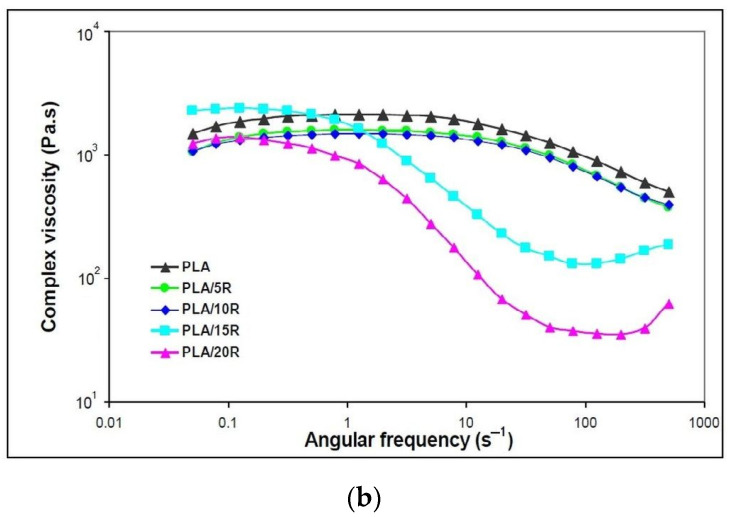
Variation of (**a**) dynamic moduli (storage moduli and loss moduli) and (**b**) complex viscosity function of angular frequency for neat PLA and blends plasticized with natural castor oil.

**Figure 7:**
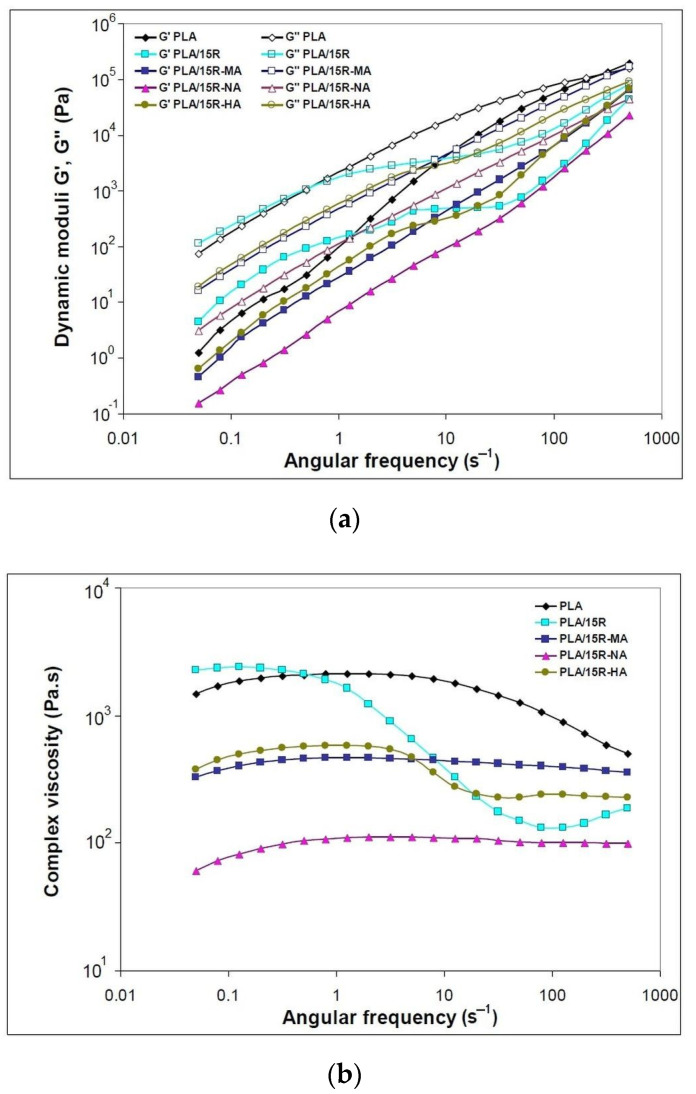
Variation of (**a**) dynamic moduli (storage moduli and loss moduli) and (**b**) complex viscosity function of angular frequency for neat PLA and blends plasticized with modified castor oil.

The dynamic rheological parameters recorded at 175 °C are representative of the variation of viscoelastic properties function of angular frequency (ω) for the neat PLA and the studied blends, namely storage modulus (G′), loss modulus (G″) and dynamic viscosity (η*) are shown in Figure 6 and Figure 7. 

The neat and plasticized PLA predominantly shows viscous behavior (G″ > G′) over all tested ω regions. G′ and G″ dependence on deformation frequency presents the same trend, with obvious differences being observed for the highest loadings of natural R.

As the storage modulus is linked to the solid-like character of a polymer, the semicrystalline PLA registered the highest G′ values, explaining also the highest melt viscosity and required power for melt processing. The shear rate is very low at lowest frequencies, with the capacity of retaining the original strength of materials being high. G′ rises with frequency because the shear rate increases as well, which also increments the amount of energy input to the polymer chains [52]. 

It is noticeable in Figure 6a that the slopes of G′ (ω) and G″ (ω) for PLA blended with the lowest amounts (5 and 10 wt%) of pure R were slightly lower than the values of neat PLA, while the highest pure R loadings of 15 and 20 wt% considerably changed the shapes of the slopes. In this latter case, both dynamic moduli, G′ and G″, present a slightly sinusoidal shape explained by a change in the material elasticity. Higher G′ values compared to neat PLA were found at low ω, up to 1 s^−1^, followed by a small “rubbery” plateau with independent G′ values function of ω and further increase of G′ values at high ω. Similar behavior has been reported for incorporating 15 wt% epoxidized soybean oil (USE) into the PLA matrix [43].

The addition of all studied amounts of natural R that act as plasticizers improved the melt flow when applying external forces, with the decrease of complex viscosity observed in Figure 6b. This behavior indicates that the PLA chains are easily deformed, decreasing their frictional resistance.

Neat PLA and PLA samples containing 5 wt% and 10 wt% natural R showed a Newtonian behavior in the frequency region up to 10 s^−1^, where the complex viscosity remains constant, followed by a shear-thinning effect to the end of testing. At high R loadings, a “full rheological flow curve” was found for PLA/15R and PLA/20R, like the PLA/USE material reported in another study [43]. At high ω, viscosity increases due to a thickening effect for high R amounts added to the PLA matrix.

When modified R was incorporated into PLA, the resulting materials’ viscoelastic characteristics changed the different functions of applied frequencies, as plotted in Figure 7a. The amplitude sweep (AS) test results for PLA were inserted in the Supplementary Materials (Figure S2). At higher ω (over 20 s^−1^), the G′ of the PLA/R-MA and PLA/R-HA samples are slightly higher than that of the PLA/natural R. In these cases; increased toughness was observed related to PLA material containing unmodified R. This is attributed to the stiff anhydrides groups of the modified R incorporated into the polymer chain. 

In the case of modified R, the Newtonian plateau is larger for PLA/R-MA and R-NA, while the sample PLA/R-HA also showed another plateau at higher ω (Figure 7b). The intermolecular interactions that occurred during blending melt led to macromolecular chains’ stabilization, and resistance to deformation could justify the plateau at higher oscillation frequencies [53]. 

The low values of complex viscosity for PLA/R-NA material could be explained by the low molecular mass of NA-modified R that improves PLA melt flow. This behavior is correlated with melt processing results (Table 2), where PLA/R-NA showed the lowest torques and power values.

### 3.6. DSC Results

**Figure 8:**
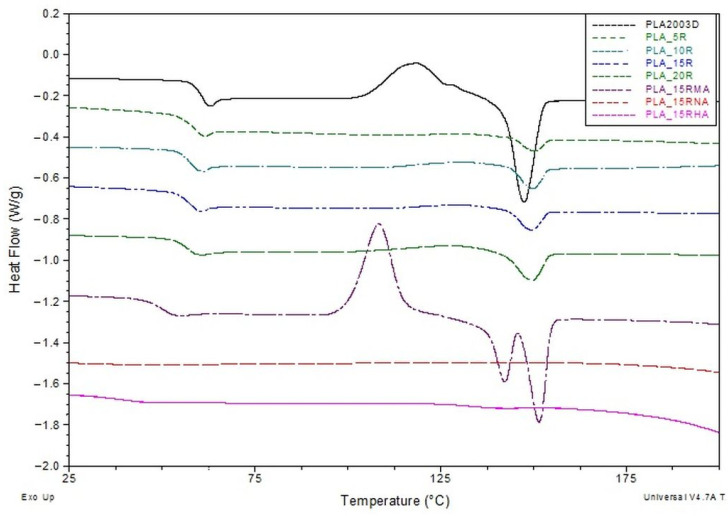
DSC curves (exo up) from the second heating for neat PLA and PLA plasticized with natural and modified castor oil.

The thermal behavior of PLA and PLA containing modified and unmodified R has been assessed from the DSC curves recorded during the second heating, after cooling from the melt state (Figure 8), since the thermal history produced during processing was erased during the first heating. The thermograms recorded during the first run and cooling (Figure S3) are inserted in the Supplementary Materials.

The samples’ glass transition (Tg) is clearly observed both on cooling following the first heating and on the second heating scans. At a first observation of the second heating DSC scans, it is clear that Tg values of PLA/R blends decrease with the addition of different amounts of R in the PLA, as compared with Tg of neat PLA. One can assume this thermal behavior is due to a plasticization effect induced by R molecules on the PLA matrix, R having a much low molecular weight compared with PLA. Castor oil contains triglycerides with long aliphatic chains that can diffuse easily among the PLA molecular chains, increasing, thus, the free volume. This behavior has been similarly observed in other studies, where PLA has been blended with various plasticizers [39,43,54,55,56,57,58].

Considering the relationship between Tg and crystallinity for a neat semicrystalline polymer, it is well-known that the crystalline regions in semicrystalline polymers constrain the amorphous phase, resulting in a reduced relaxation that implies relatively higher Tg values for the sample with higher Xc. Therefore, we consider this plasticization effect more important than the constraint effect imposed by the crystallinity on amorphous phase relaxation. When a fraction of 15 wt% maleinized R (15R-MA) is added to the PLA matrix, a further decrease of Tg value (to 48.94 °C) is noted, most likely occurring due to an additional increase of the free volume of PLA induced by MA molecule (the space is bigger than that occupied by aliphatic chains from unmodified R), that might lead to an increase of plasticization effect (Table 3). PLA containing R modified with NA or HA does not show any measurable glass transition or melting process during second heating in DSC curves. However, both transitions were evident in the first run (see Figure S3a in Supplementary Materials). The absence of the melting peak for PLA/15R-NA and PLA/15R-HA samples on the second heating DSC curves could be assigned to a strong crystallizability reduction of PLA chains due to the molecular structure of NA and HA that imbedded the PLA chain packing and might require more time to complete the crystallization process.

The cold crystallization is usually denoted in a DSC curve by a clear exothermic process with a sharp shape, limited to a narrower range. In the case of our samples, the cold crystallization process is clearly observed for neat PLA, but no such thermal event is recorded when R is added, regardless of content. We assume that even if a weak but extended exotherm over a wide temperature range is slightly noticeable in the DSC curves of PLA/R samples in Figure 8, it cannot be attributed to cold crystallization. The absence of a cold crystallization could be explained by a dramatic change in PLA viscosity when R is added.

Regarding the melting process of PLA-based samples, it can be observed that DSC curves show only a clear endothermic process without any shoulder or some noticeable modification in the shape. The melting temperatures in Table 3 indicate slight increasing values for PLA samples containing R with the same T_m_, regardless of content. The less sharpened DSC melting endotherms alongside the slightly higher Tm values compared with neat PLA denote an improvement of PLA chain mobility through R diffusion. This increased mobility causes an interruption of PLA chain packing, resulting in a less number of crystals with lower dimensions but slightly more thermodynamically stable. The melting enthalpy usually results from two crystal types: one type obtained after the sample processing that crystallizes on cooling, and a second type achieved during the cold crystallization process during reheating. According to Equation (3), the crystallinity degree (Xc) is evaluated after subtracting the cold crystallization enthalpy from the melting enthalpy value. The resulting value is representative only of the crystal fraction developed during melting. According to the data in Table 3, one can mention that the overall crystallinity index increases with R content, related to neat PLA, supporting thus the plasticization effect induced by R incorporated in PLA. These results agree with the I_crystalline_/I_amorphous_ ratio of PLA/R blends from Figure 3.

The plasticization of PLA by R-MA incorporation seems to have a different effect on chain mobility. DSC curves record a strong cold crystallization process of PLA/15R-MA sample at lower temperatures. This behavior denotes that PLA chains are arranged into a more packed (ordered) structure at this temperature. Still, the crystallinity index decreases when 15 wt% of R-MA is added, compared with PLA containing 15 wt% unmodified R [59]. R likely becomes a more rigid molecule through maleinization, thus proving its role in nucleation and facilitating PLA chain packing at lower temperatures.

Regarding the PLA/15R-MA melting, one can notice that a double endotherm represents this process, usually ascribed to the mechanism of the sample’s melting, crystallization and re-melting behavior. Other studies have also reported this behavior, which is explained by the different sizes and perfection of crystallites as a consequence of lamellar rearrangements during PLA crystallization [60,61,62]. The melt temperature of the second endotherm DSC peak is higher (with 4 °C) than even of neat PLA. This result further demonstrates that the R-MA acted as a nucleating agent in addition to the plasticizer role for PLA, leading to more perfect PLA crystals but fewer in number.

### 3.7. Thermal Stability and Degradation

**Figure 9:**
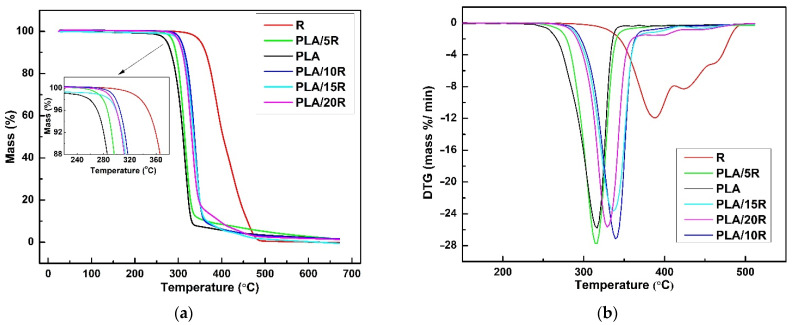
TG (**a**) and DTG (**b**) thermograms of castor oil (R), neat PLA and PLA/R blends.

**Figure 10:**
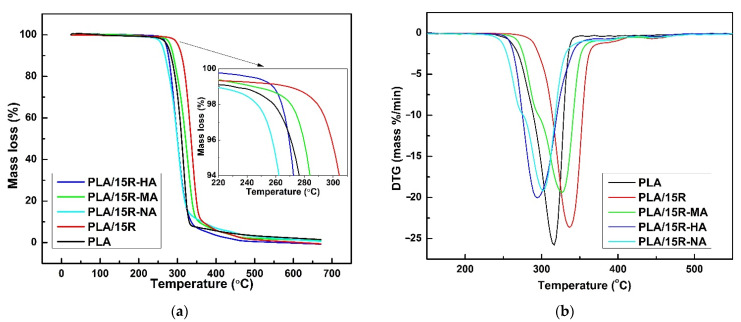
TG (**a**) and DTG (**b**) thermograms of neat PLA and PLA containing 15 wt% modified castor oil.

To acquire the characteristics of a plasticizer for PLA, the candidates should exhibit low volatility and be thermally stable at or slightly above the PLA melting temperature, conditions imposed by processing. The thermal degradation behavior of neat PLA and its blends with R unmodified and modified with anhydrides were investigated by thermogravimetric analysis, and the results are shown in Figure 9 and Figure 10. 

The thermal degradation parameters such as T_onset_ (starting temperature of decomposition), T5% and T30% (the temperatures corresponding to 5 wt% and respectively 30 wt% mass losses) from the TG curve, as well as Tmax (the temperature at which the degradation rate is maximum) from the DTG curve obtained for neat PLA and its respective blends, were summarized in Table 4, that also included parameters for R modified with MA, NA and HA, previously determined by Mustata and Tudorachi from the TG curve as well as Tmax (the temperature at which maximum degradation occurs) from the DTG curve obtained for neat PLA and its respective blends were summarized in Table 4, that also included parameters for R modified with MA, NA and HA, previously determined by Mustata and Tudorachi [37].

The heat-resistant index (Ts), which indicates the overall thermal stability of PLA blends, has been calculated by using Equation (4) [63]. Hafiezal et al. refer to the heat-resistance index as the temperature of the polymer in the physical heat tolerance limit [64].

Ts = 0.49 [T5% + 0.6(T30% − T5%)] (4)

DTG curve of natural R (Figure 9b) showed three partially overlapped steps of mass loss assigned to decomposition and/or volatilization of different compounds in various temperature ranges. R thermally decomposed in three consecutive stages; in the first one, several highly volatile and unstable compounds (oxygenated compounds, hydroperoxides, and polyunsaturated fatty acids) undergo decomposition, according to other results [65]. The second phase refers to the decompositions of ricinoleic acid (monounsaturated fatty acid), while the decomposition of saturated fatty acids takes place in the third stage. 

Thermal decomposition of PLA matrix involved the cleavage of functional groups, including C-C, C-O, and C=O bonds. The weight loss for PLA is recorded between 265–380 °C, being attributed to the thermal degradation due to the trans-esterification reaction that cleaves the PLA backbone structure [66]. 

It can be seen both from the TG/DTG curves plotted in Figure 9 and the data shown in Table 4, respectively, that natural R is more thermally stable than PLA-containing ester groups. Therefore, the shifts to higher values of T_onset_ (Figure 9a, inserted graph)_,_ T_max,_ and T_s_ show the improvement of the thermal stability of PLA/5R and especially PLA/10R sample, compared to neat PLA. This enhancement in thermal stability was also recorded for higher R fractions incorporated in the PLA matrix (PLA/15R, PLA/20R), with slightly lower thermal parameters related to PLA/10R. However, a higher thermal resistance when compared with PLA. The explanation of this behavior could be provided by R decomposition, which in the second stage of its mechanism involves an increase in ricinoleic acid degradation rate, leading to PLA matrix degradation. Moreover, this behavior is clearly noticeable in Figure 9b, where the second stage of the R decomposition mechanism becomes more evident for PLA matrix decomposition in samples PLA/15R and PLA/20R. As shown in Table 4, the mass loss value assigned to each second or third-stage decomposition mechanism for these samples is not negligible, which further supports our assumption. The DTG curves (Figure 9b) show that the sample containing 10 wt% R proves the highest thermal stability. But for this content of R and lower one (5 wt%), the rate of the degradation mechanism of PLA matrix slightly increases, a maximum mass loss being recorded. 

The obtained results regarding the R decomposition are similar to the ones reported by Mustata and Tudorachi in their work [37], where thermal degradation of R modified with three types of anhydrides has been investigated. According to the reported thermal degradation kinetic parameters, the order of thermal stability of R modified with cyclic anhydrides is R-HA > R-NA > R-MA. By melt blending of PLA with each of this modified R in a fraction of 15 wt%, the thermal stability of all samples decreased compared to the PLA blended with the same fraction of unmodified R (Figure 10). 

According to Figure 10a and data presented in Table 4, the thermal stability decreases more for PLA/15R-NA but seems to be the only sample containing modified R with such behavior. One can say that a different diffusion of molecules/radicals resulting during degradation (water, alcohols, saturated and unsaturated hydrocarbons, carbon monoxide and dioxide, and carbonyl compounds) could affect the rate of the overall mechanism. Based on the DTG curves shown in Figure 10b, it can be noted that the decomposition mechanism for PLA blended with modified oils occurs at a slightly slower rate compared to pure PLA or PLA containing 15 wt% unmodified R.

As shown by the data obtained by Mustata and Tudorachi [37], the most important percentage of mass loss during the degradation of R resulted in the first step when the aliphatic chains were broken (C-C bonds). Analyzing the data obtained in this study, it can be seen that R shows the lowest percentage of residue after the degradation mechanism compared to the blended samples. Although R increases the thermal stability of the PLA matrix (increasing the T_onset_ value), the overall degradation process becomes more dynamic with increasing of natural R content. The intermediate compounds resulting between the degradation steps (II and III) led to an acceleration of the degradation mechanism. This hypothesis is clear in the results from the percentage of residues at the end of the degradation process, as shown in Table 4. In contrast, PLA samples containing modified R show a reduction in the residue values, indicating a higher degradation mechanism intensity.

## 4. Conclusions

Castor oil unmodified (up to 20 wt% of the total blend) or modified with three cyclic anhydrides (15 wt% of the total blend) could be fully incorporated into the PLA matrix to improve the melt processing, mechanical, thermal, and rheological properties of neat PLA. 

An exponential decrease of processing parameters was observed when increasing the amount of natural R in PLA from 5 to 20 wt%, as the frictional resistance is reduced due to much easier deformation of the PLA chains, improving melt flow and consequently melt processability. A rise in the processing parameters has been recorded for PLA/15R-MA and PLA/15R-HA when compared with PLA/15R, possibly due to the physical interaction between the PLA matrix and anhydride-containing plasticizers.

Both natural and modified R blended with PLA lowered the surface wettability of the resulting materials, increasing their surface hydrophobicity.

The mechanical testing results demonstrated enhanced flexibility for PLA/natural R materials and a reinforcing effect for PLA blended with modified R in relation to PLA containing 15 wt% pure R, with both strength at break and Young modulus values increasing. Among the PLA/modified R blends, incorporating R-MA offered the best tensile properties for the respective PLA blend.

The samples containing unmodified R showed increased thermal stability, while those containing modified R changed the dynamic of the overall degradation mechanism, PLA/15R-MA showing the best onset thermal stability from all modified R.

Based on the obtained data, plasticized PLA with natural R could be recommended for the manufacturing of flexible films, while those containing anhydride-modified R could be used for semi-rigid food packaging.

## Figures and Tables

**Table 1:** Labeling, composition and visual aspects of neat PLA and PLA-based materials containing unmodified and modified castor oil.

Sample	PLA (wt%)	Natural Castor Oil (wt%)	Modified Castor Oil (wt%)	Visual Aspect
PLA	100	0	0	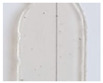
PLA/5R	95	5	0	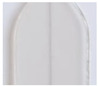
PLA/10R	90	10	0	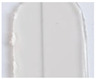
PLA/15R	85	15	0	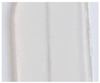
PLA/20R	80	20	0	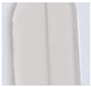
PLA/15R-MA	85	0	15	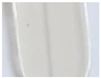
PLA/15R-NA	85	0	15	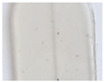
PLA/15R-HA	85	0	15	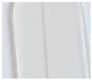

**Table 2:** Processing characteristics for neat and plasticized PLA.

Sample	TQ_max_ (Nm)	TQ_1min_ (Nm)	TQ_5min_ (Nm)	TQ_fin_ (Nm)	Melt Viscosity (Pa·s)	Power (kW)
PLA	70	65	37	28	0.467	0.176
PLA/5R	28.9	7.1	6.9	5.8	0.097	0.036
PLA/10R	18.7	6.2	5.3	4.8	0.080	0.030
PLA/15R	13.9	4.1	2.2	2.1	0.035	0.013
PLA/20R	12.7	2.8	1.4	1.4	0.023	0.009
PLA/15R-MA	10.7	3.7	3.1	2.5	0.042	0.016
PLA/15R-NA	12.1	3.4	2.3	1.1	0.018	0.007
PLA/15R-HA	10.3	2.6	2.4	2.3	0.038	0.014

**Table 3:** Thermal parameters resulted from the second heating DSC scans of neat and plasticized PLA.

Sample	Tg (°C)	Tcc (°C)	∆Hcc (J/g)	Tm (°C)	∆Hm (J/g)	X_c_ (%)
PLA	59.82	118.48	17.66	147.43	19.65	2.12
PLA/5R	57.60	-	-	150.42	2.101	2.37
PLA/10R	56.93	-	-	150.08	3.376	4.00
PLA/15R	56.71	-	-	149.84	3.274	4.11
PLA/20R	56.26	-	-	149.91	5.161	6.88
PLA/15R-MA	48.94	108.42	25.21	141.96; 151.48	27.98	3.48

**Table 4:** Thermogravimetric data of neat PLA and PLA/R blends.

Sample	T_onset_ (°C)	T_max_ (°C)	∆W_step_ (%)			Residue (%)	T_5%_ (°C)	T_30%_ (°C)	T_s_ (°C)
			I	II	III				
PLA	264.56	316.15	98.46	-	-	1.54	273.50	301.00	142.10
PLA/5R	281.41	324.00	98.53	-	-	1.47	286.40	305.80	146.04
PLA/10R	303.56	338.80	98.41	-	-	1.60	305.12	327.20	156.00
PLA/15R	285.86	335.20	91.15	3.93	5.53	0.62	299.80	324.60	154.19
PLA/20R	298.11	328.90	85.91	7.29	5.74	1.12	302.70	322.50	154.14
R	344.33	388.00	57.09	31.17	11.97	0.23	351.60	385.70	182.30
MA *		426(III)	14.61	21.15	61.51	3.15	166.00	438.00	161.00
NA *		434(III)	14.79	28.84	54.8	0.62	184.00	425.00	161.00
HA *		423(III)	5.73	35.83	57.15	1.29	213.00	425.00	167.00
PLA/15R-MA	271.40	327.00	91.74	3.50	3.79	0.97	256.70	286.70	134.60
PLA/15R-NA	250.68	300.30	88.76	5.03	5.54	0.68	283.20	306.20	145.53
PLA/15R-HA	260.93	294.20	94.55	6.13	-	0.75	270.50	290.30	138.37

* Reprinted with permission from Ref. [37]. 2022, Elsevier.

## Data Availability

The data presented in this study are available on request from the corresponding author.

## Supplementary Materials

The following supporting information can be downloaded at: https://www.mdpi.com/article/10.3390/polym14173608/s1, Figure S1: Torque-time curves for the neat PLA and PLA plasticized with natural and modified castor oil. Figure S2: Amplitude sweep test results for PLA; Figure S3: DSC curves (exo up) from the first heating (a) and cooling (b) for neat PLA and PLA plasticized with natural and modified castor oil.
