# Supplementary material for: Evaluation of Natural and Modified Castor Oil Incorporation on the Melt Processing and Physico-Chemical Properties of Polylactic Acid
Source: Polymers (Basel). 2022 Sep 1;14(17):3608. doi: 10.3390/polym14173608 (PMC9460240; doi:10.3390/polym14173608)
Supplement: Supplementary file 1 [file polymers-14-03608-s001.zip › polymers-1890471-supplementary.pdf]

polymers

Supplementary Materials

Evaluation of Natural and Modified Castor Oil Incorporation on the Melt Processing and Physico-Chemical Properties of Polylactic Acid

Raluca Nicoleta Darie-Niță [1], Anamaria Irimia [1], Vasile Cristian Grigoraș [1], Fănică Mustață [1], Niță Tudorachi [2], Maria Râpă [3,*], Joanna Ludwiczak [4] and Andrzej Iwanczuk [4]

[1] Physical Chemistry of Polymers Department, Petru Poni Institute of Macromolecular Chemistry, 41A Grigore Ghica Voda Alley, 700487 Iasi, Romania
[2] Natural Polymers, Bioactive and Biocompatible Materials Department, Petru Poni Institute of Macromolecular Chemistry, 41A Grigore Ghica Voda Alley, 700487 Iasi, Romania
[3] Faculty of Materials Science and Engineering, University Politehnica of Bucharest, 313 Splaiul Independentei, 060042 Bucharest, Romania
[4] Faculty of Environmental Engineering, University of Science and Technology, 50-013 Wrocław, Poland
* Correspondence: maria.rapa@upb.ro

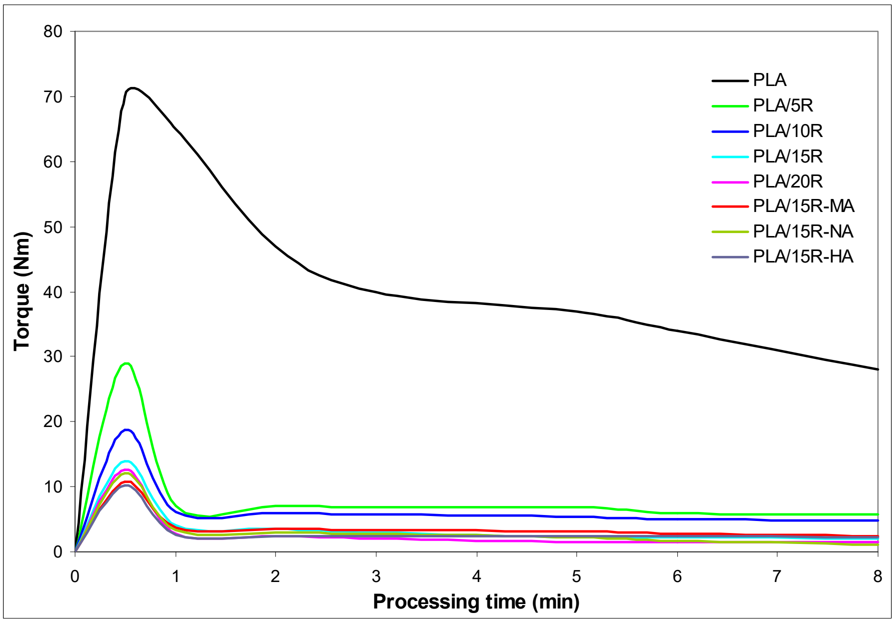

Figure S1. Torque-time curves for the neat PLA and PLA plasticized with natural and modified castor oil.

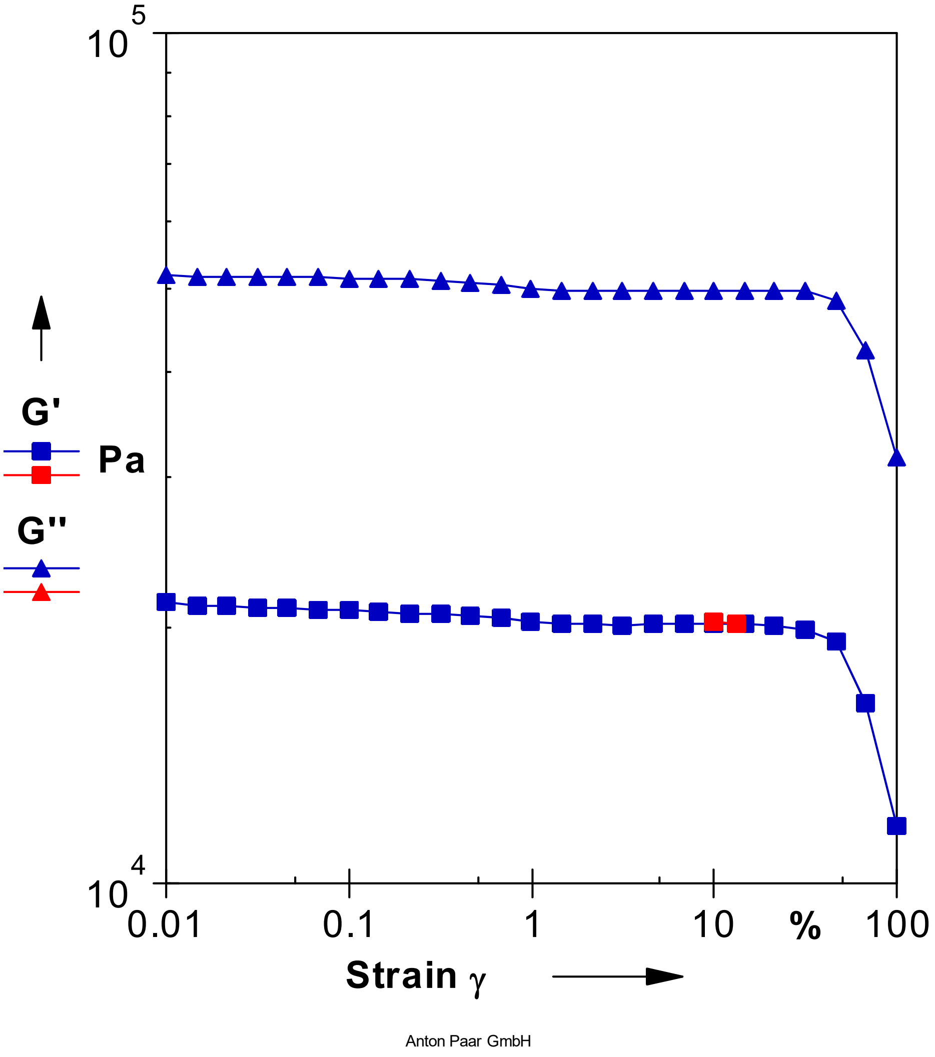

Figure S2. Amplitude sweep test results for PLA.

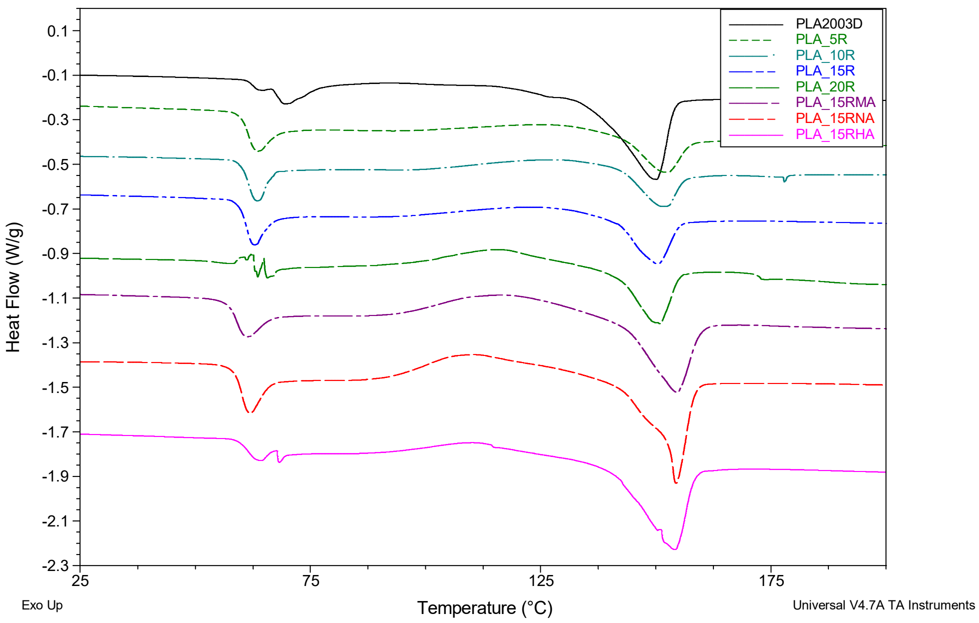

(a)

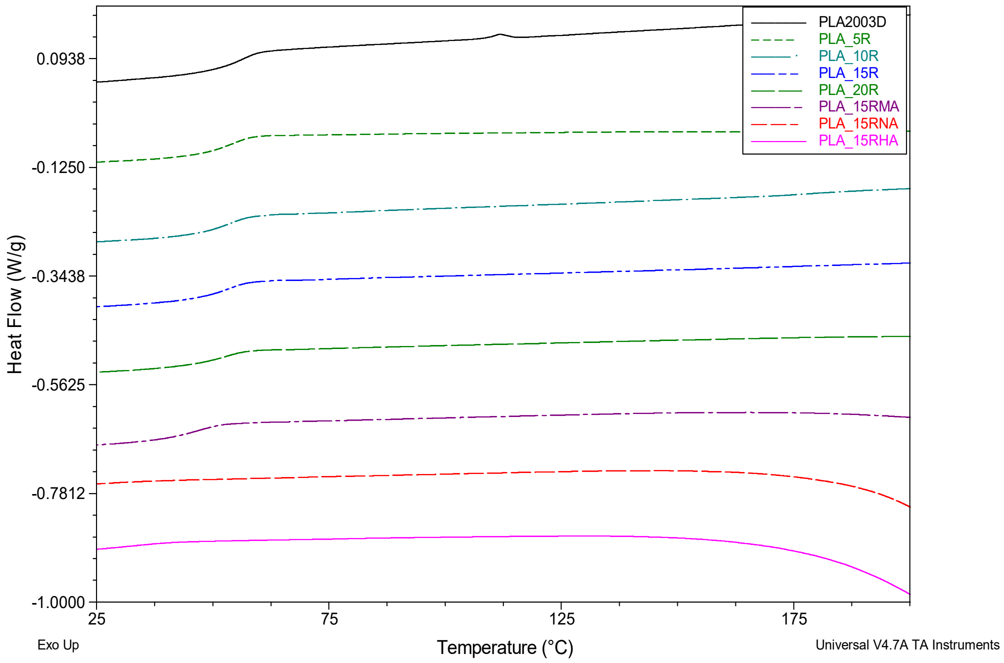

(b)

Figure S3. DSC curves (exo up) from the first heating (a) and cooling (b) for neat PLA and PLA plasticized with natural and modified castor oil.
